# Acute type A dissection with malperfusion: Immediate central repair and complication-specific strategies

**DOI:** 10.1016/j.xjtc.2026.102488

**Published:** 2026-06-22

**Authors:** G. Chad Hughes, Jovan Markovic

**Affiliations:** Divisions of Thoracic and Cardiovascular Surgery and Vascular and Endovascular Surgery, Department of Surgery, Duke University Medical Center, Durham, NC

**Figure:**
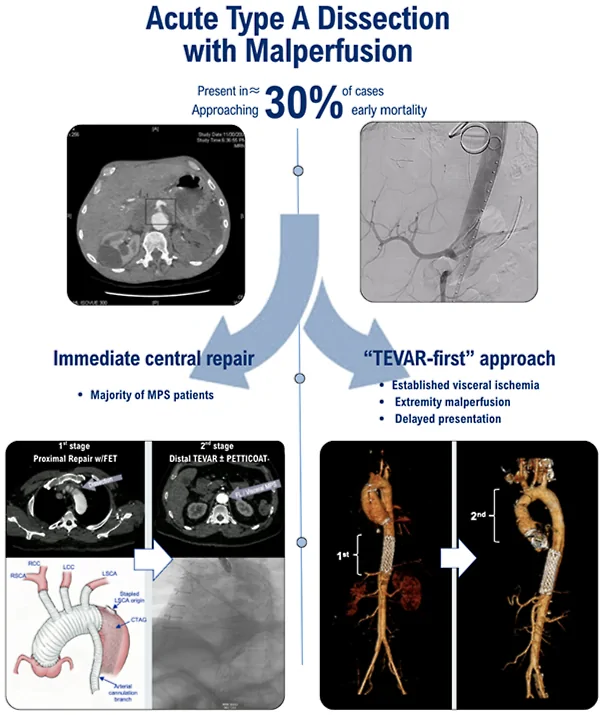
Institutional algorithm for ATAD w/malperfusion: central repair versus TEVAR-first approach.

**Central Message**

We favor an algorithmic approach to acute type A dissection with malperfusion, with immediate central repair for most patients and complication-specific strategies reserved for established ischemia.

Recent data from the Society of Thoracic Surgeons Adult Cardiac Surgery Database examining nearly 10,000 patients undergoing repair of acute type A dissection (ATAD) demonstrate that preoperative malperfusion is present in nearly 30% of cases and is associated with an early mortality rate approaching 30%.^1^ Accordingly, ATAD complicated by malperfusion syndrome (MPS) remains one of the most formidable challenges in contemporary cardiothoracic surgery.

Considerable controversy persists regarding the optimal management strategy for these patients. Some centers advocate immediate central aortic repair in all patients regardless of malperfusion status,^2^ whereas others favor a complication-specific approach^3^ with initial transcatheter intervention followed by delayed central repair. High-level comparative data between these strategies are lacking, and randomized trials are unlikely, given the acuity and heterogeneity of presentation. At our institution,^4^ management is therefore algorithmic rather than dogmatic: immediate central repair is favored in the majority of patients with MPS, whereas a staged, complication-specific strategy is reserved for select patients with established visceral ischemia or delayed presentation with extremity malperfusion in whom immediate central repair would pose unacceptable risk. Importantly, this delayed or staged strategy requires careful patient selection. Hemodynamic instability, rupture or tamponade physiology, severe aortic insufficiency with heart failure, coronary malperfusion, or other proximal life-threatening features generally mandate immediate central repair. Cerebral malperfusion is considered separately according to mechanism and neurologic severity on a case-by-case basis.

In this Invited Case Video, we present 2 representative cases that illustrate both approaches (Video 1). The first case involves a patient with acute B_0_ dissection with a primary entry tear in the aortic arch, retrograde intramural hematoma extending into the ascending aorta, and distal dissection with marked true lumen compression throughout the visceral segment. Importantly, the patient demonstrated radiographic-only malperfusion without clinical evidence of end-organ ischemia. The patient was treated with ascending aortic replacement and zone 2 total arch replacement with antegrade frozen elephant trunk deployment, followed by staged completion thoracic endovascular repair using the PETTICOAT (Provisional Extension to Induce Complete Attachment) technique 2 days later to fully address distal true lumen compression. This case highlights that in the absence of irreversible end-organ injury, immediate central repair effectively resolves dynamic malperfusion in most patients.

The second case involves a patient with previous mechanical aortic and mitral valve replacements who presented with ATAD complicated by established visceral MPS because of a delayed diagnosis. At presentation, the patient had peritonitis and radiographic evidence of irreversible bowel ischemia, rendering immediate central repair unsafe. Importantly, the patient also had no immediately life-threatening proximal complication mandating central repair. Thus, this case represents an appropriate example of patient selection for a delayed, complication-specific strategy: distal malperfusion was the dominant life-threatening problem, whereas immediate redo central repair would have posed prohibitive risk. The patient therefore underwent emergent right colectomy followed by a complication-specific “thoracic endovascular repair-first” strategy, a technique first described by our group in 2010,^5^ in which a thoracic endograft is deployed in the compressed true lumen of the distal descending thoracic aorta to relieve dynamic visceral MPS. The patient experienced a prolonged postoperative course related to complications of bowel resection but ultimately recovered and underwent successful redo sternotomy with ascending aortic and hemiarch replacement several months later.

Together, these cases underscore the importance of tailoring operative strategy to the mechanism, severity, and timing of malperfusion in ATAD. Although immediate central repair remains the preferred approach for most patients, selective use of staged, complication-specific strategies is essential in those with established end-organ injury or delayed presentation.

Institutional review board approval and patient consent were not required for this report.

## Conflict of Interest Statement

G.C.H. reported consultant, speaker, and scientific advisory board for W. L. Gore & Associates J.M. reported no conflicts of interest.

The *Journal* policy requires editors and reviewers to disclose conflicts of interest and to decline handling or reviewing manuscripts for which they may have a conflict of interest. The editors and reviewers of this article have no conflicts of interest.

## Supplementary Data

Video 1 Operative and imaging illustration of 2 acute type A dissections with malperfusion, one managed by immediate central repair with staged completion thoracic endovascular repair and one by a complication-specific thoracic endovascular repair-first strategy after emergent colectomy. Video available at: https://www.jtcvs.org/article/S2666-2507(26)00295-6/fulltext.
